# Influence of gut microbiota on eye diseases: an overview

**DOI:** 10.1080/07853890.2021.1925150

**Published:** 2021-05-27

**Authors:** Pasquale Napolitano, Mariaelena Filippelli, Sergio Davinelli, Silvia Bartollino, Roberto dell’Omo, Ciro Costagliola

**Affiliations:** Department of Medicine and Health Sciences “V. Tiberio”, University of Molise, Campobasso, Italy

**Keywords:** Microbiota, gut, eye disease, dysbiosis, probiotics

## Abstract

The microbiota is a dynamic ecosystem that plays a major role in the host health. Numerous studies have reported that alterations in the intestinal microbiota (dysbiosis) may contribute to the pathogenesis of various common diseases such as diabetes, neuropsychiatric diseases, and cancer. However, emerging findings also suggest the existence of a gut-eye axis, wherein gut dysbiosis may be a crucial factor influencing the onset and progression of multiple ocular diseases, including uveitis, dry eye, macular degeneration, and glaucoma. Currently, supplementation with pre- and probiotics appears is the most feasible and cost-effective approach to restore the gut microbiota to a eubiotic state and prevent eye pathologies. In this review, we discuss the current knowledge on how gut microbiota may be linked to the pathogenesis of common eye diseases, providing therapeutic perspectives for future translational investigations within this promising research field.

## Introduction

The human gut microbiota comprises more than 100 trillion microorganisms associated with multiple functions, from nutrient metabolism to protection against pathogens [1]. The gut microbiota has a metabolic activity similar to that exhibited by other organs of human body [2]. Each individual encompasses a unique gut microbiota profile that changes over time, depending on certain variables such as lifestyle, physical exercise, body mass index (BMI), and cultural and dietary habits [3]. Although in the past, it was considered just as a passive passenger, the intestinal microbiota is now considered as a complex and dynamic ecosystem that contributes to the proper functioning of body's immune system and maintenance of the health state [4]. Since 2007, an initiative launched and funded by the National Institutes of Health, called “The Human Microbiome Project” [5]. has identified reference genomes, describing the microbiota of healthy human hosts [6,7]. Advances in sequencing technology, especially 16S rRNA sequencing, have played a key role in determining the composition of microbiota, which has been found to be mainly dominated by Proteobacteria, followed by Actinobacteria, Firmicutes, and Bacteroidetes. Several studies have found that various eye diseases are associated with gut and dysbiosis, broadly defined as an intestinal microbial imbalance in the composition of resident commensal communities relative to the community found in healthy individuals. These variable regions of highly conserved 16S rRNA genes make it possible to distinguish bacterial genera from each other.

Although controversial, the existence of a gut-eye axis has also been demonstrated in ophthalmology. Intestinal microbiota appears to be essential in propagating inflammatory diseases of the eye [8]. and could represent a potential target for further approaches in the treatment of severe and chronic ocular conditions, as manipulation of the gut microbiome has been shown to influence the course of ocular diseases. Scientists have focussed their attention on how microbial components can influence human health by enhancing the proliferation of beneficial microbes. For example, *Lactobacillus* can decrease the number of neutrophil extracellular traps. *B. fragilis* shows protective effects against autoimmune diseases through its polysaccharide capsule. Based on these assumptions, ophthalmologists have highlighted the advantages of the direct use of probiotics and prebiotics in clinical practice. However, this area of research needs to be better explored. Prebiotics, defined as short-chain carbohydrates, have shown a positive influence in restructuring gut immunity and gut barrier function as metabolic substrates for *Lactobacillus* and *Bifidobacterium* species. In addition, their combined use with probiotics, defined as live microbial components, increases the modulation of gut immunity. Nevertheless, the mechanisms involved in regulation of the gut-eye axis have not yet been completely clarified.

## Autoimmune uveitis

**Figure 1.**
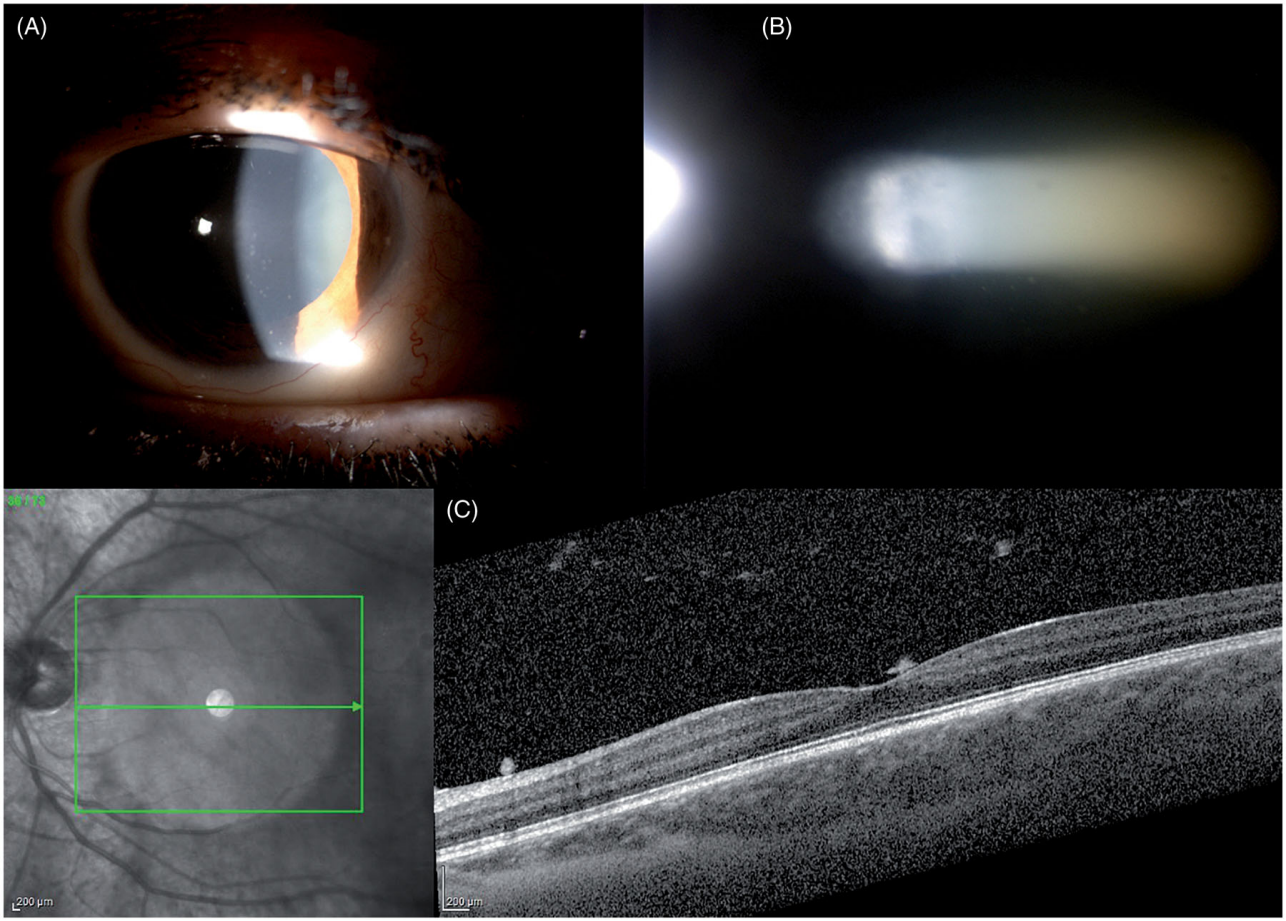
Autoimmune Uveite. (A) Endothelial depositsvand and iris nodules; (B) Flare and Tyndal; (C) SD-OCT Vitreitis.

Uveitis, defined as flogosis of the uvea, is a disease that can cause blurred vision and requires appropriate treatment. Its classification is based on anatomic position (anterior, intermediate, and posterior), aetiology (infectious, non-infectious, autoimmune, and drug induced), and course (acute, recurrent, and chronic) [9,10]. The prevalence of uveitis is 5.4 per 1000 people in the USA and presents a direct correlation with age and smoking [11]. Most of the uveitis cases are non-infectious and idiopathic autoimmune. In contrast, infectious cases represent a small minority of the total cases observed. Acute anterior uveitis (AAU) is one of the most common forms, representing 85% of the total cases observed and is related to the leukocyte cell surface protein–human leukocyte antigen B27 (HLA-B27) (Figure 1). Recently, evidence has linked the microbiome and uveitis owing to clinical observations regarding the association of diet with chronic uveitis. The first study, conducted using animal models, compared HLA-B27-positive transgenic rats and negative littermate controls. A significant difference in the intestinal bacterial composition between the groups was recorded [12]. Rosenbaum et al. first focussed on the potential activation mechanism of the interaction between the microbiome and uveitis [13]. Their study hypothesised four mechanisms of activation. In the first one, they hypothesised that dysbiosis causes an alteration in intestinal homeostasis, increase in permeability, and a loss of immunity, which subsequently leads to the migration of bacterial products or activated immune cells to remote sites. Furthermore, microbiome dysbiosis can induce alterations in local intestinal immune homeostasis. This leads to lower activation of immune cells and promotes a pro-inflammatory response. Moreover, molecular mimicry processes causing reduction in tolerance towards ocular antigens that are physiologically sequestered behind the blood–ocular barrier can activate the immune system. This situation seems to be a paradox because retinal antigens are not represented in periphery; therefore, peripheral retina-specific T cells that are found circulating must be activated to enter the eye and cause the infection [14]. Lin et al. [15]. compared the microbiota of transgenic rats for HLA-B27 and human b2-microglobulin to the microbiota of wild-type controls, using 16S rRNA gene sequencing. It was found that disruption of barrier function enables the migration of microbial products and immune cells into the eye [16]. Since a long time, various animal models of uveitis have been used for understanding human uveitis. Experimental autoimmune uveitis (EAU), one of the most commonly used uveitis models, was induced by active immunisation with the retinal protein interphotoreceptor retinoid-binding protein (IRBP) emulsified in complete Freund’s adjuvant (CFA), which is a mixture of mineral oil, and heat-killed *Mycobacterium tuberculosis* (MTB). In particular, the study highlighted the enrichment of *Prevotella*, *Lactobacilli*, and *Clostridium* species two-week post-immunisation. In contrast, control animals showed a relative enrichment of intestinal *Ruminococcus* and *Proteobacteria* species. Horai et al. [17]. observed a delayed onset of uveitis in germ-free transgenic mice and transgenic mice treated with a broad-spectrum oral antibiotic. Moreover, the induction of germ-free gut in animal models caused uveitis [18]. Oral administration of broad-spectrum antibiotics (metronidazole, vancomycin, neomycin, and ampicillin) determined variations in the gut microbiome and attenuated uveitis. This antibiotic cocktail is usually used to reduce gut bacterial load [19]. Although metronidazole and ampicillin are well-absorbed from the intestinal tract and become systemically available, both vancomycin and neomycin are not absorbed from the gut and directly affect the microbiome load. Heissigerova et al. [20]. observed that germ-free mice were experimentally preserved from uveitis activation, similar to that observed with oral antibiotic therapy. However, unlike that in the experimental models of this disease, autoimmune uveitis can be associated with immune presentation of ocular antigens owing to their seizure behind a tight blood–retinal barrier in the healthy eye.

At the molecular level, genetic polymorphisms can alter protein function as well as the reinforced form of how epigenetic modifications regulate gene transcription regulation. This explains how environmental signals can determine genomic and epigenomic modifications to model the cellular functional output. Environmental triggers determine lasting changes to the cell when extracellular signals are translocated to the nucleus, leading to epigenetic reprogramming. This clarifies the importance of identifying the extracellular microenvironmental factors that trigger epigenetic changes, resulting in impaired cell function. Indeed, environmental factors triggering inflammation can lead to epigenetic changes responsible for intraocular inflammatory diseases such as uveitis [21]. Recent studies have shown that the onset of disease in animal models is mediated by epigenetic changes. The transcription factors Tbx21 and Rorc are known to be the main regulators of the differentiation of lymphocytes into T helper and T reg cells [22,23]. Hypomethylation of DNA factors discovered in the retinas and retinal pigment epithelium (RPE)–choroidal tissues of EAU mice was associated with an increased production of Th1/Th17-specific cytokines [interferon (IFN)-γ and interleukin (IL)-17] [24]. Additionally, miRNA-223 upregulation was also detected in this model, which was capable of promoting inflammation through the activation of T cells and myeloid dendritic cells. Furthermore, altered serum levels of miRNA-223 have been reported to be directly associated with microbiome dysbiosis. Comparisons of serum miRNA profiles between cases and controls revealed the absence of specific miRNAs associated with uveitis. These miRNAs are linked to flogistic signalling cascades, such as mitogen-activated protein kinase (MAPK), forkhead box (FOXO), and vascular endothelial growth factor (VEGF) [25].

Although these findings highlight a correlation between gut microbiota and the development of autoimmune uveitis, the underlying mechanism remains unclear. The first hypothesis formulated in this regard proposed that dysbiosis can increase intestinal permeability by facilitating the presentation of microbial products that trigger ocular inflammation, both through direct effects on the eyes and indirectly through mechanisms of molecular mimicry and immune sensitisation. Furthermore, the injection of T cells cultured from these transgenic mice into wild-type mice caused uveitis. However, this was only observed when T cells were cultured in the presence of intestinal extracts. Similar to the results of other studies, Nakamura et al. [26]. found that the induced autoimmune uveitis in mice had different profiles of intestinal commensals compared to the controls. Moreover, oral antibiotics reduced the severity of uveitis symptoms by increasing the activity of Treg cells. Therefore, they concluded that supplementation with short chain fatty acids (SCFA) decreases the severity of uveitis, reduces the effects of T cells, and increases the activity of Tregs. These results highlight strong associations between the gut microbiota and autoimmune uveitis, although further studies are needed to expand the understanding of the precise mechanism responsible for the association.

## Age-related macular degeneration (amd)

**Figure 2.**
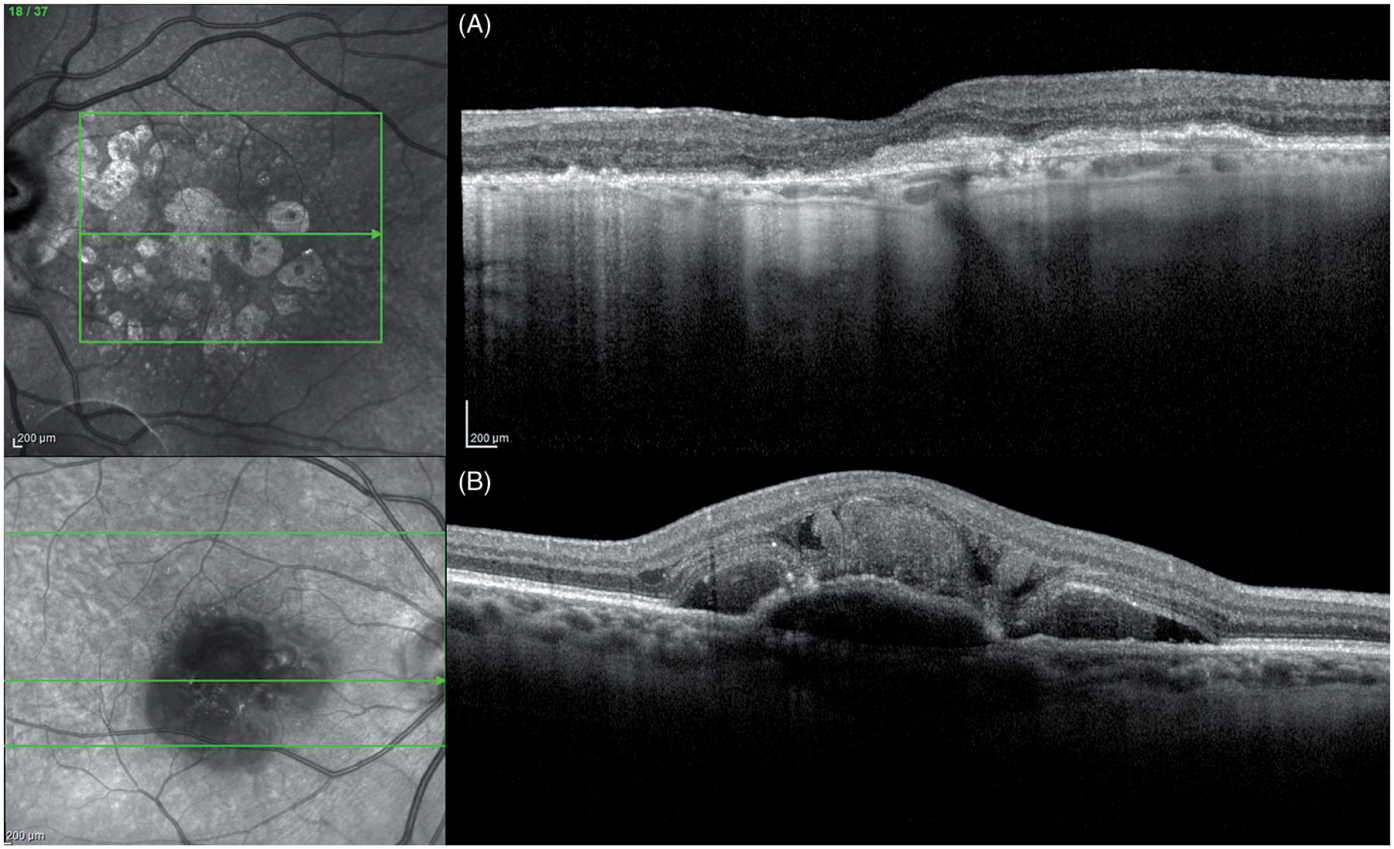
SD-OCT dry-AMD (A); SD-OCT wet-AMD (B).

Age-related macular degeneration (AMD) is a degenerative disorder that leads to a reduction in central vision. This disease preferentially affects the macular region of the retina. More than 50 million people suffer from AMD globally, with a future projection of 300 million by 2040 [27]. It is a polygenic disease with environmental influences that can be classified into dry or wet (neovascular) forms. Dry AMD can develop into wet AMD. In dry AMD, drusen and cellular debris accumulate beneath the RPE and Bruch’s membrane [28,29]. The presence of drusen could damage the RPE, leading to indirect photoreceptor cell damage. In wet AMD, choroidal neovascularization (CNV) is observed, which can cause haemorrhage, lipid exudates, and subretinal and/or intra-retinal fluid accumulation, leading to RPE detachment and ultimately RPE cell death [30]. The proliferation of CNV in wet AMD has been related to enhanced vascular and immune responses (Figure 2). Although the exact pathogenesis of AMD remains poorly understood, some inflammatory mechanisms associated with innate immunity have been identified. A previous study showed that wild-type C57Bl/6 J mice fed long-chain fatty acids and C3 (found in high glycemic index diets) developed clinical signs of AMD, such as degeneration of photoreceptors, lipofuscin accumulation, hypopigmentation, and RPE atrophy [31]. however, these changes were not observed in control mice of the same age and sex. Furthermore, the pathophysiology of dry AMD includes accumulation of lipofuscin in the EPR layer, which promotes retinal oxidative damage. Mitochondrial defects cause intracellular and extracellular toxin accumulation, similar to that in neurodegenerative diseases [32]. Moreover, complement system dysregulation can induce inflammatory cell damage and the activated immune cells tend to infiltrate microglia and macrophages. The activation of some leucine-rich repeat-containing family- and pyrin domain-containing 3 (NLRP3)-mediated inflammasomes causes the release of IL-1β and IL-18 in mouse retinal tissues due to drusen induction [21]. Oral antioxidant supplementation according to AREDS2 is the only intervention that appears to slow the progression of AMD. Animal studies have shown that zinc intake improves antioxidant processes in the retina by reducing oxidative stress [33]. Especially, the intestinal bacterial flora competes for the supply of zinc, which is useful both for symbiotic metabolic pathways and for bacterial virulence factors [34]. However, both zinc deficiency and its presence in excess can alter the composition of the microbiome. The relationship between microbiota and oral supplementation was verified by Lin [8]. Patients with AMD showed an increased number of *Prevotella* spp. compared to the controls. Furthermore, he found a reduction in the abundance of *Ruminococcaceae* and *Rikenellaceae* bacteria. Another interesting finding was that *Oscillobacter*, *Anaerotruncus*, and *Eubacterium* species were associated with increased gut permeability and inflammatory changes (increased levels of IL-6 and IL-8). Moreover, patients with AMD showed elevated levels of *Streptococcus* and *Gemella* species and reduced levels of *Prevotella* and *Leptotrichia* species when compared to the controls [35]. thus enabling bacterial products and pathogen-associated molecular patterns (PAMPS) to enter the circulation and interact with downstream pattern recognition receptors (PRRs). The interaction between microbiota and toll-like receptors (TLRs) activates the innate immune response. In relation to this, gut microbiome dysbiosis is associated with chronic inflammation and can increase intestinal permeability. The intestinal levels of several intestinal bacteria, most predominantly *Peptoniphilius*, are influenced by integration with AREDS vitamins, which increases in patients with AMD taking AREDS. In a study by Lin, patients with AMD presented higher levels of *Ruminococcaceae* and *Prevotella* than the controls. Some experimental models have demonstrated that intestinal microbiome variation also exacerbates CNV, at least in part, in human models. Experimental mice were used to correlate high glycemic index diet, obesity, and oxidative stress with the progression of dry AMD to wet AMD. Skondra et al. demonstrated that a high-fat diet worsens the severity of dry and wet AMD in animal models when genetic predisposition is present. Rowan et al. [36]. instead found that a high glycemic index (HG) diet determines histological characteristics of dry AMD, as opposed to models fed a low glycemic index (LG) diet. They found that both the composition and metabolic activity of the gut microbiota were different between the two groups. Detailed analysis of their work by evaluating the retinal damage measuring the outer retinal layer (ONL) thickness revealed decreased thickness in HG models in contrast with that in LG models. Moreover, they estimated the worst damage progression in the former model. In addition, they focussed on the slowed progression of damage when the HG model was switched to the LG model. Supporting this hypothesis, Zinkernagel et al. [35]. highlighted that individuals with AMD had a relative abundance of *Anaerotruncus*, *Oscillibacter*, and *Ruminococcus torques* with degradation abilities, and *Eubacterium ventriosum*, whereas *Bacteroides eggerthii* was enriched in the controls. They concluded that a high-fat diet exacerbated CNV in humans and was related to an increase in *Firmicutes* in the gut. These findings were associated with a reduction in tight junction strength and induction of inflammatory cytokines such as IL-6, IL-8, IL-1β, TNF-α, and VEGF-A [37,38]. These cytokines are related to the development of neovascular AMD. Several studies on the microbiome and AMD have focussed on post-translational modifications, in particular, histone acetylation and DNA methylation in the retina [21, 39]. Some sequences of hypermethylation in patients suffering from AMD were the glutathione S-transferase P1 (GSTP1) promoter, which is related to a reduction in mRNA expression of the two isoforms of GSTM (GSTM1 and GSTM5). GSTM proteins reduce the levels of reactive oxidative species, which in turn reduces retinal oxidative damage. In fact, hypermethylation can increase retinal oxidative stress. In contrast, AMD patients present a characteristic hypomethylation of the IL-17 receptor C (IL17RC) promoter, causing an increase in its expression. This receptor is a promoter of the inflammatory cascade. Moreover, histone deacetylation limits the accumulation of clusterin, a protein produced by RPE, the major constituent of drusen. Thus, we propose that the gut microbiota associated with the influence of its metabolites can be considered as a biomarker for AMD and can be potentially targeted for therapeutic modulation of the disease.

## Primary open angle glaucoma (POAG)

Glaucoma is a disease characterised by progressive irreversible optic neuropathy related to damage to retinal ganglion cells (RGCs). The primary clinical feature is the loss of the peripheral visual field extending to the centre, which in the terminal stages is able to determine a complete loss of vision [40]. The incidence of primary glaucoma is constantly growing [41]. Glaucoma is defined as an optic neuropathy related to an increase in intraocular pressure (IOP) and alteration of drainage of aqueous humour in the anterior chamber of the eye. In open-angle glaucoma, an open drainage method is not sufficient to maintain normal IOP. Furthermore, the outflow pathways are dysfunctional, causing an increase in the IOP. Long periods of high increased pressure are related to mechanical impairment, ischaemia, oxidative stress, and inflammation within the optic nerve [42]. This condition is related to extracellular matrix remodelling of the lamina cribrosa and optic nerve head [43–45]. A deep study of literature allowed us to detect many factors that could be linked to POAG, such as age, systemic diseases, hypotension or hypertension, diabetes, hyperlipidaemia, thyroid disease, obstructive sleep apnoea, and genetic mutations in the *MYOC*, *CYP1BI*, *FOXC1*, *PITX2*, *PAX6*, and *OPTN* genes [46,47]. Few studies have investigated the association between gut glaucoma and the microbiota. In particular, they focussed on mitochondrial DNA in patients with glaucoma [31].

Two variants of bacteria have been reported to be associated with POAG and alterations of the intestinal bacterial flora, particularly with respect to the relative abundances of *Bacteroides* and Prevotella [31,33]. Furthermore, a high number of nuclear genes has been linked to POAG, many of which are involved in mitochondrial function [48]. For example, POAG lymphoblasts present a complex I defect of the oxidative phosphorylation pathway. This leads to a decreased rate of respiration, which can confer an increased susceptibility to retinal ganglion cell damage [49]. Other authors identified many mitochondrial functions that are protective against glaucomatous optic neuropathy. Shibuya et al. [34]. focussed on the correlation between normal tension glaucoma and polymorphism of TLR4, which plays a key role in the recognition of bacterial lipopolysaccharide (LPS). It is widely known that the activation of TLR4 stimulates the production of inflammatory cytokines, activating the innate immune system by decreasing the rates of lipolysis and beta-oxidation. This stops the functioning of bacterial pyruvate and acetyl-CoA by limiting their growth [8]. In addition, glaucomatous patients show an upregulation of TLR4. In these patients, the expression of TLR4 was prominently located in retinal microglia cells. Unfortunately, the link between gut microbiome alterations and their pro-inflammatory effects related to TLR4 polymorphisms in glaucoma is unknown. Zinkernagel et al. [35]. highlighted that peripheral injection of bacterial LPS stimulated axonal degeneration and neuronal decrease in two separate animal models of glaucoma. This effect was probably related to TLR4 upregulation and consequent activation of the complement and damage of the retinal and optic nerve microglia. Scientists have tried to evaluate whether bacterial lysates can improve glaucomatous neurodegeneration. For this purpose, low-dose subcutaneous bacterial LPS was administered to two separate glaucomatous mouse models. The study model allowed us to find a link between the peripheral administration of LPS and increased activation of microglia in the optic nerve, related to the loss of RGCs. Thus, bacteria activate axonal microglia to promote neurodegeneration. Gupta [37]. instead suggested that the intestinal microbiota stimulates the production of neuroprotective factors, promoting the survival of RGCs. The same research team also highlighted a possible relationship between glaucoma and *Helicobacter pylori* [36,38]. More precisely, a higher rate of *H. pylori* infection was found in patients with glaucoma and the normal tension controls [50]. Some mechanisms have been proposed to explain this correlation, such as cytokines, ureases, and the neutrophil-activating protein VacA [51]. This inflammation is the activator of immune cells. These effects could activate microglia and lead to their differentiation into phagocytic macrophages in the optic nerve [52,53]. A recent meta-analysis by Zeng et al. [54]. linked *H. pylori* to an increased risk of normal tension glaucoma and open-angle glaucoma. In contrast, there was no association between secondary glaucoma and pseudoexfoliation syndrome. They supposed a migration of reactive oxygen species and inflammatory cytokines from the gastric mucosa to the optic disc or trabecular meshwork [55]. with a possible cross-reactivity of *H. pylori* IgG antibodies with ocular tissues [56]. On the other hand, an intraocular *H. pylori* colonisation was expected to be present, as shown by the presence of this bacterium in the histology of trabeculectomy specimens [57]. Nevertheless, the involvement of *H. pylori* in the pathogenesis of glaucoma remains controversial, with large differences in the diagnostic criteria of the various studies. Another possible relationship was deduced by Astafurov et al. [58]., who proposed a link between oral bacteria (e.g. *Streptococci*) and a worse oral microbial composition in patients affected by glaucoma compared with sane controls. Moreover, in a study named “Health Professionals Follow-up study” the authors found a relationship between tooth loss and glaucoma diagnosis [59]. Thus, chronic and low-grade inflammation caused by oral dysbiosis is a trigger of glaucomatous damage. At the molecular level, epigenetic changes have been linked to optic nerve damage. Epigenetic changes in retinal ganglion cells may also be related to alterations in glaucoma. This epigenetically altered homeostasis could be related to alterations in the gut microbiota. Thus, flogosis caused by microbial dysbiosis could activate microglial regulation by: (1) direct bacterial dissemination to the optic nerve and/or retina, (2) bacterial product dissemination to the optic nerve, (3) affecting secondary to vascular system alteration, and (4) variations observed in the systemic immune system.

## Dry eye and contact lens wearing

**Figure 3.**
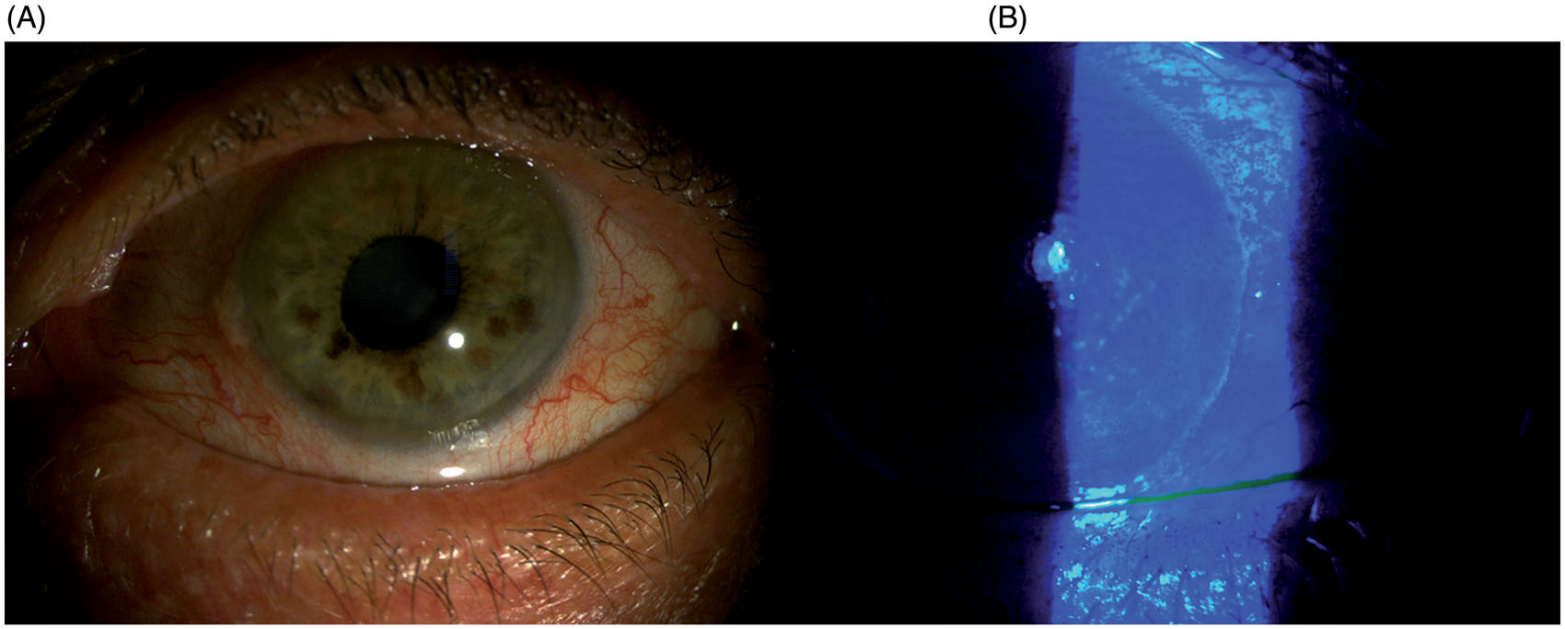
Slit lamp picture of dry eye disease (A); Alteration of fluorescein distribution and reduction of BUT (B).

Dry eye (Figure 3) is a multifactorial disease of the tear and the ocular surface. The main symptoms are discomfort, visual disturbance, and tear film instability with potential damage to the ocular surface [58]. Ocular surface is a complex system composed of the cornea, conjunctiva, meibomian glands, lacrimal glands, and the neural network. One of the other components that should be considered is the microbiome. Ocular surface protection is guaranteed by several types of immune cells. As we already know, ocular surface-associated lymphoid tissue (EALT) is composed of mucosal immune systems associated to conjunctiva (CALT) and lacrimal drainage tissue. Activation of the ocular surface immune system is regulated by innate and adaptive immune systems [60]. which are mediated by T cells and antibodies secreted by the plasma cells [61,62]. Microbiota has not been considered crucial in the regulation of this homeostasis. Nevertheless, a recent study by Dong et al. [63]. characterised all bacterial and viral components of the ocular surface microbiota defining local immune tolerance and microbe representation similar to that of the gut microbiome. Moreover, alterations in the microbiome status of the ocular surface are related to alterations in homeostasis. Even a single component diminution can alter this homeostasis, triggering dry eye disease (DED) and promoting the vicious cycle of the disease. Ocular microbiota impairment is evident in chronic contact lens users and DED. This alteration in microbiota activates the innate immune response. In a recent study, every component of ocular surface microbiota was detected, showing the prevalence of *Staphylococcus aureus* and coagulase-negative *Staphylococcus*, *Corynebacterium*, and *Propionibacterium* in patients suffering from DED [64]. Kountouras et al. [65]. found an alteration in the composition of ocular microbiome influencing ocular autoimmunity and reducing IgA production. Studies have shown that CD-25 knockout mice spontaneously develop the Sjögren’s dry eye phenotype. Furthermore, this alteration was linked to a variation in microbial composition [66]. Other studies demonstrated that germ-free CD25 knockout mice improved their dry eye symptoms after a faecal microbiota transplant (FMT) [67]. On the contrary, a recent study by Jiang et al. [68], exploring the composition of the ocular microbiome in patients with Meibomian gland dysfunction (MGD), observed a reduction in coagulase-negative Staphylococcus abundance despite the microbial ocular surface composition of healthy people [69]. Moreover, *S. aureus* and *Klebsiella* spp. were abundantly present on the ocular surface of patients with MGD [70,71].

Several studies have suggested that osmolarity (OSM) alterations, seen in dry eye and blepharitis, could be related to gut microbiota dysregulation. In addition, they explored the bacteria found in Meibomian gland secretions in an effort to find a stronger relationship between microbial composition and MGD severity. Data collected by the authors were based on clinical signs, such as conjunctival injection, upper and lower tear meniscus height (TMH), tear breakup time (TBUT), corneal staining, lid margin, orifice, tear foam, and Meibomian gland assessment. According to the literature, the commensal microbiome changes with the severity of MGD. In particular, the abundance and complexity of the microbiome have increased. However, the low-mild pattern of MGD presented an increased number of bacterial species compared to the control, as *Microbacteriaceae* and *Bacillus* were missing. On the contrary, in mild MGD, the percentage of bacterial population was higher than that in the control, which indicated an altered microenvironment. One of the most representative samples detected in the severe MGD group was *Corynebacterium*, particularly *C. macginleyi*. In contrast, *S. epidermidis* was found in the normal controls. In conclusion, Bonini et al. found a relationship between vernal keratoconjunctivitis and probiotic eye drops [72]. In their study, they administered *Lactobacillus acidophilus* eye drops in patients with Vernal keratoconjunctivitis, prepared using freeze-dried inactivated *L. acidophilus*, four times a day in both eyes for one month after three days of washout. They demonstrated that a 4-week treatment relieved the signs and symptoms of vernal keratoconjunctivitis in the active phase. Thus, we can assert that many associations exist between the microbiota and ocular surface disease, and further studies are needed to characterise the precise mechanisms responsible for these associations.

## Chalazion

Chalazion is a subacute or chronic inflammation of the meibomian gland. It is defined as the simultaneous or subsequent occurrence of multiple chalazia, which can be sporadic or recurring. Several factors are involved in the pathogenesis of chalaziosis—constitutional atopic and seborrhoeic dermatitis; hormonal imbalance; immunological factors; irritable bowel disease; iatrogenic infections [73,74]. (especially related to *S. aureus and Propionibacterium acnes*); demodicosis (Demodex mite infestation) [75]. and dysmetabolic factors (e.g. vitamin A deficiency and diabetes) [76,77]. Moreover, excessive intake of saturated fats can result in a change in the composition of the lipids secreted by the meibomian glands. The secreted fluid loses its fluidity and it is difficult to spill it over from the gland, which ultimately leads to chalazia formation. Moreover, disruption of the normal ocular surface microbiome could have a significant role as a cofactor in the pathogenesis [78]. Exposure to various environmental factors, including diet, toxins, drugs such as antibiotics, and pathogens, can impair microbiome homeostasis, leading to dysbiosis [79]. As already described by Kugadas et al., exposure of the host to gut commensal species may serve as a priming signal to generate B cell repertoires at sites different from the gut, such as eye-associated lymphoid tissues. Further explanation may be that microRNAs (miRNAs), which are noncoding small RNAs, are important epigenetic regulators implicated in pathologic signalling and are found extracellularly in different body fluids. miRNAs operate in a posttranscriptional manner and are crucial for several biological events. Recently, the authors speculated on possible crosstalk between miRNAs and microbiota. Supporting evidence of miRNAs has already been emphasised in some recent studies [39]. Interestingly, altered serum levels of miRNA-223 have been linked to microbiota dysbiosis, and an upregulation of miRNA-223 was detected in the autoimmune uveoretinitis rat model [25]. Therefore, it is realistic to hypothesise a role for miRNAs in various ocular diseases. Regarding chalaziosis, it is plausible to suppose an association among probiotics, miRNAs, and changes in the composition of the secreted fat from the Meibomian glands, which makes it less fluid. Several studies have shown that probiotics could play a role in the prevention and treatment of different diseases in children. Filippelli et al. [80]. demonstrate that probiotic supplementation reduces the time taken for complete resolution of the chalazion without inducing noteworthy complications and prevents its recurrence in the treated children. Probiotic supplementation was performed using a mixture of live *S. thermophilus*, *Lactococcus lactis*, and *L. delbrueckii* subsp. Bulgaricus. In particular, they are gram-positive, anaerobic bacteria, which produce lactic acid and other antimicrobial substances, such as hydrogen peroxide and bacteriocins (ribosomal synthesised antimicrobial peptides with bactericidal effects) [81]. In conclusion, oral probiotic supplementation has a favourable influence on the clinical course of one of the most common eye disorders, at least for small lesions, without inducing noteworthy complications. In our previous study [80]., we found that supplementation with probiotics is a safe and effective therapy for reducing chalaziosis recurrence.

## Probiotics and prebiotics

Scientists have focussed their attention on how microbial components can influence human health to enhance the proliferation of beneficial microbes [82]. Lactobacilli, for example, are able to decrease the number of neutrophil extracellular traps [83]. *B. Fragilis* provides protective effects against autoimmune disease through its polysaccharide capsule [84]. To our best knowledge, results from meta-analyses are equivocal [85]. given that ophthalmologists have the advantage of directly using probiotics in their clinical practice. However, this area of research needs to be explored further. Through a thorough literature search, we were able to find some studies evaluating the stability of an eye drop probiotic formulation containing *S. boulardii* and *L. rhamnosus* in patients with vernal keratoconjunctivitis. Itching, photophobia, burning, and tearing improved significantly at two weeks and four weeks, with a statistically significant improvement in clinical signs. Interestingly, they reported downregulation of TLR4, which was also observed after four weeks of treatment [86]. An additional consideration should be given to several therapeutic strategies targeting gut microbiota for the treatment of ocular disease. One of the proposed approaches is targeting the causative bacteria, in an effort to use more specific antibiotics or designed immunoglobulins that target individual causative bacteria. The latter was explored by Okai et al. [87]. A second approach targeting gut microbiota directly through oral administration of live bacterial strains guarantees immune homeostasis by enhancing Treg differentiation, although this probiotic approach would need to be designed with pre-clinical experiments [88,89]. A final approach may be to supplement an entire community of intestinal bacteria with a normal community using FMT.

Recently, various studies have investigated how prebiotics can improve the course of ocular diseases. Prebiotics are defined as non-digestible foods that have beneficial effects on human health, influencing the activity and growth of probiotics in the colon after fermentation [90]. Since their first study, prebiotics have been used to manipulate microorganisms and their interactions with human health. During the first year of study, prebiotics were recognised only as booster of the growth of bifidobacteria and lactobacilli. Subsequently, they were recognised for their metabolic and physiological functions [91]. Filippelli et al. identified probiotics not only as predominately carbohydrate-based foods but also as polyphenols and polyunsaturated fatty acids. Low-molecular-weight carbohydrates represent a metabolic substrate for *Bifidobacteria*. They possess extracellular glycosidases and specific transport systems, guaranteeing their rapid assimilation of low-molecular-weight sugars. The metabolism of polysaccharides to short chain fatty acids (SCFA) is a complex pathway that ends with acetate and lactate, the metabolic end products of *Bifidobacteria* [92]. Starting from these studies, it is easy to understand that the gut response to carbohydrates is heavily influenced by the composition of intestinal microbiota. For example, individual Prevotella-dominant microbiota can ferment carbohydrates more rapidly than the Bacteroides-dominant microbiomes [93]. Given that, we have a clear understanding of the ecology of the gut microbiota, and discovering the mechanisms of action of prebiotics represents a challenge to interact with it. Despite this issue, we can postulate probable mechanisms of influence. The premise is that prebiotics entering the gut are selectively metabolised. This condition can determine the growth and functionality of local bacteria that influence human health. Immune regulation can be influenced by increased cell wall components and metabolic products, such as organic acids, which lower intestinal pH and affect microbial pathogens and mineral absorption. Furthermore, these metabolic products can influence epithelial integrity and hormonal regulation, thereby reflecting immune system modulation. All of these postulated mechanisms are supported by *in vitro* or animal studies, although in many cases, it is difficult to establish that they occur within the human gut microbiota.

## Conclusion

recently, there has been increasing interest in the interaction between eye and gut microbiota research in ophthalmology. The role of the microbiome is slowly beginning to emerge. The studies we reported here attest that ocular and extraocular microbiota contribute to some ophthalmic diseases. These studies confirmed the presence of a gut–eye axis, ocular infections, and inflammatory conditions. The mechanisms underlying these associations have recently become clearer. However, a number of critical barriers and different interpretations have been found to prove the role of microbioma in individual pathologies, and the temporal relationship, whether specific bacteria/viruses/fungi are linked to pathology compared to normal controls. Future innovation in this field may lead to a new target in ophthalmology to understand and manage ophthalmic diseases, providing alternative or adjunctive local or systemic treatments to modulate the ocular surface and gut microbiota.
